# Phenological Stage and Nitrogen Input Coordinately Regulate Bud Bank Dynamics and Shoot Allocation in an Alpine Clonal Perennial Grass

**DOI:** 10.3390/plants14142164

**Published:** 2025-07-14

**Authors:** Keyan He, Qingping Zhou, Lin He, Lili He, Haihong Dang, Xiaoxing Wei, Qian Wang, Jiahao Wang

**Affiliations:** 1 Qinghai Academy of Animal Science and Veterinary Medicine, Qinghai University, Xining 810016, China; keyan1961006779@163.com (K.H.); 18893651860@163.com (L.H.); 13073197156@163.com (L.H.); wuiko@163.com (X.W.); 2 Laboratory for Germplasm Research and Utilization on the Qinghai-Tibet Plateau, Qinghai University, Xining 810016, China; 3 College of Grassland Resources, Southwest Minzu University, Chengdu 610041, China; 4 State Key Laboratory of Sanjiangyuan Ecology and Plateau Agriculture and Animal Husbandry (Co-Founded by the Ministry and Province), Qinghai University, Xining 810016, China; wangqian@qhu.edu.cn; 5 Institute of Grassland Research, Chinese Academy of Agricultural Sciences, Hohhot 010010, China

**Keywords:** belowground bud bank, nitrogen addition, phenological stage, shoot allocation, Qinghai–Tibet Plateau

## Abstract

Belowground buds play a vital role in the clonal propagation and structural regulation of perennial herbaceous plants, especially in alpine environments, where vegetative renewal depends heavily on bud bank dynamics. However, the interactive effects of nitrogen addition and phenological stages on bud development and aboveground branching remain poorly understood. In this study, we examined the responses of rhizome buds, tiller buds, and aboveground tiller types of Kentucky bluegrass to six nitrogen levels (0, 6, 9, 12, 15, and 18 g/m^2^) across five growth stages on the Qinghai–Tibet Plateau. The results showed that moderate nitrogen input (N2, 9 g/m^2^) significantly enhanced total bud density, particularly at the heading and maturity stages, indicating a threshold response. Aboveground reproductive tiller density peaked at N2 (9 g/m^2^), while vegetative and total tiller densities plateaued beyond N3 (12 g/hm^2^), suggesting a diminishing marginal effect of nitrogen on aboveground tiller density. Furthermore, bud density showed stage-specific correlations with tiller types: vegetative tillers were primarily influenced at the heading stage, and reproductive tillers were mainly influenced at the mature stage, with weakened associations in senescence. These findings highlight the phenological specificity and non-linear response of clonal grass regeneration to nitrogen input and provide a theoretical basis for optimizing nutrient management in cold alpine grasslands.

## 1. Introduction

Belowground buds—such as rhizome buds and tiller buds—serve as critical structures for individual regeneration, population renewal, and community stability in perennial herbaceous plants [1,2]. Compared with seed-based reproduction, belowground buds exhibit several adaptive advantages, including rapid renewal capacity, high survival rates, and strong spatial plasticity [1,3,4]. These advantages are particularly essential under harsh environmental conditions, such as those in alpine [2,5] or arid regions [6,7], where these adaptations play an irreplaceable role in sustaining plant persistence and spatial expansion. In addition to serving as the foundation for vegetative propagation, belowground buds regulate the formation of both vegetative and reproductive tillers, thereby contributing to the balance between vegetative growth and reproductive investment [8]. Vegetative tillers support photosynthesis, while reproductive tillers are responsible for sexual reproduction; both are significantly influenced by bud density and bud type.

In alpine regions such as the Qinghai–Tibet Plateau, vegetation renewal is highly dependent on bud activity [9,10]. The influence of belowground buds on aboveground structures exhibits strong phenological specificity [5], with distinct differences in bud activation and utilization across developmental stages [11]. For instance, during the early to mid-growth stages (such as the heading stage), plants tend to prioritize the activation of vegetative tillers to expand photosynthetic area, whereas in later stages (such as maturity), resources are more likely to be allocated to reproductive tillers to ensure seed development and dispersal [11,12]. As plants enter senescence, the functions of aboveground organs begin to decline, and belowground buds gradually lose activity and enter dormancy [5,13,14,15], leading to a weakened correlation between the bud bank and aboveground structure.

Nitrogen is a key limiting nutrient for plant primary productivity and organ development, directly influencing branching patterns and bud differentiation [16,17]. In perennial grasses, nitrogen availability can alter the differentiation rate and survival probability of belowground buds by regulating internal carbon-to-nitrogen (C:N) balance, hormonal dynamics, and metabolic activity [18,19], thereby shaping the development of vegetative and reproductive tillers [20,21]. Previous studies have demonstrated that nitrogen addition not only enhances aboveground biomass accumulation [22,23] but also plays a critical role in modulating plant resource allocation strategies, reproductive modes, and community structures [24]. However, the mechanisms by which different nitrogen levels regulate the formation and functional expression of belowground buds remain unclear, particularly with regard to how bud banks respond to nitrogen inputs across phenological stages and contribute to aboveground-structure differentiation. An increasing amount of empirical evidence suggests that plant responses to nitrogen are not linearly positive but rather follow a pattern of a diminishing marginal effect [25]: while a moderate amount of nitrogen promotes growth, excessive inputs may suppress organ development or trigger physiological stress. This phenomenon is especially critical for perennial clonal plants that rely on bud bank dynamics for regeneration, as an over-supply of nitrogen may disrupt the balance between bud dormancy and activation [5,26], ultimately affecting the temporal and spatial stability of the bud bank.

Numerous studies have demonstrated the effects of nitrogen addition on biomass accumulation and community composition in perennial grassland ecosystems [27,28,29]. Nevertheless, our mechanistic understanding of how nitrogen regulates belowground bud differentiation and activation to coordinately drive aboveground structural development remains limited. This knowledge gap is particularly pronounced in alpine regions, where perennial clonal grasses rely heavily on bud banks for regeneration. The relationship between belowground buds and the development of vegetative and reproductive tillers is shaped by complex environmental gradients and the phenological rhythms in such environments, yet an integrated theoretical framework is still lacking. Cold-adapted perennial grasses such as Kentucky bluegrass (*Poa pratensis* var. anceps) exhibit strong bud bank regulatory traits, with the spatiotemporal dynamics of bud density and type serving as key drivers of tiller formation and functional expression. As a representative cool-season perennial grass, Kentucky bluegrass plays a vital ecological role in grassland establishment and renewal across the Qinghai–Tibet Plateau [30]. However, clear evidence is still lacking on how different levels of nitrogen addition affect bud bank dynamics and, more importantly, how these dynamics regulate tiller differentiation and resource allocation patterns across phenological stages. In particular, the interactive effects of nitrogen gradients and plant developmental stages on belowground–aboveground structure formation have long been overlooked, limiting both theoretical advancements and the practical application of nitrogen management strategies in the sustainable development of alpine artificial grasslands.

Therefore, this study focuses on Kentucky bluegrass and examines how belowground bud density and aboveground tiller types respond to multiple nitrogen addition levels across successive phenological stages. The following hypotheses were proposed: (1) The density of belowground buds is co-regulated by nitrogen input and plant phenology. A moderate nitrogen level will markedly enhance bud density, particularly during the heading and maturity stages, indicating stage-specific responsiveness to optimal nutrient availability. (2) According to the law of diminishing marginal returns, tiller density will peak under moderate nitrogen input conditions, whereas it will tend to plateau when there are high nitrogen levels. (3) The regulatory role of belowground bud density in tiller development will exhibit strong phenological specificity, with vegetative tillers being most responsive during the heading stage, reproductive tillers being the most responsive during the maturity stage, and both showing weakened associations during the senescence stage. In summary, this study aims to reveal the regulatory mechanisms and phenology-dependent relationships between belowground bud bank dynamics and aboveground shoot allocation under nitrogen gradients. The findings are expected to provide a theoretical foundation for optimizing nitrogen management strategies in artificial grasslands in cold regions and offer practical guidance for improving the tillering structure, productivity, and clonal regeneration capacity of perennial forage grasses, with important ecological significance and applicational value.

## 2. Materials and Methods

### 2.1. Experimental Materials and Site Description

The field experiment was located at Qinghai University’s Haibei Experimental Station (36°49′29.7″ N, 101°45′26.6″ E; elevation 3150 m), situated on the northeastern Qinghai–Tibet Plateau. An artificial Kentucky bluegrass pasture was initially established in 2018 on a 59 m × 40 m plot. The average annual temperature in this area is 1.81 °C, with extreme maximum and minimum temperatures of 22 °C and −20 °C, respectively. The mean annual precipitation is 380 mm, primarily concentrated between July and September, and the annual evaporation figure is approximately 1650 mm. The area receives about 2980 h of sunshine annually. Soil properties in the 0–30 cm layer were as follows: pH, 8.33; total nitrogen, 1.52 g/kg^1^; total phosphorus, 1.35 g/kg^1^; soil organic carbon, 20.01 g/kg^1^; hydrolysable nitrogen, 2.44 mg/kg^1^; available phosphorus, 1.28 mg/kg^1^; and available potassium, 165.79 mg/kg^1^.

An artificial Kentucky bluegrass grassland was established in May 2017 using a manual row-seeding method. Seeds with a germination rate above 88% were used, all of which were provided by the Qinghai Academy of Animal Science and Veterinary Medicine. Xining, Qinghai, China. The seeding rate, set in accordance with the research by Wei [31], was 3 g/m^2^, with a sowing depth of 2 cm and a row spacing of 30 cm. After establishment, the grassland was maintained under natural conditions, with manual weeding conducted once per year.

### 2.2. Experimental Design

**Figure 1:**
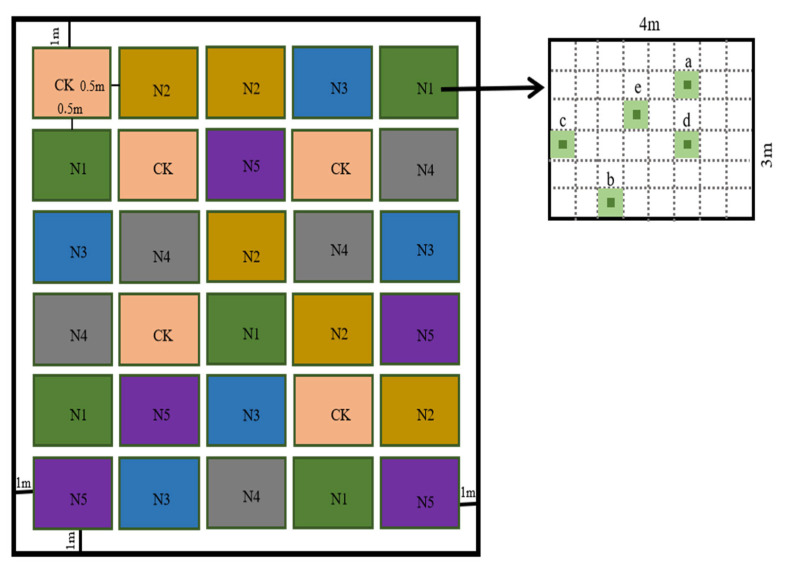
Layout of treatments in the experimental block: a—7 July (heading stage); b—25 July (flowering stage); c—10 August (dough stage); d—21 August (mature stage); and e—5 September (senescence stage). The light green squares (0.5 m × 0.5 m) represent plant community survey quadrats, while the dark green central squares (0.25 m × 0.25 m) indicate the bud bank sampling subplots placed at the center of each vegetation quadrat.

A nitrogen addition experiment was conducted in the fifth year after the establishment of the Kentucky bluegrass pasture in Qinghai Province. Following the nitrogen application guidelines for alpine grasslands proposed by Zhang et al. [32] and Yang et al. [33], five nitrogen application levels were established, namely, 6, 9, 12, 15, and 18 kg/hm^2^, designated as N1, N2, N3, N4, and N5, respectively. A no-fertilizer treatment (CK) served as the control. The experiment had a completely randomized block design with five replicates per treatment. Each block measured 3 m × 4 m, with 0.5 m buffer zones between adjacent blocks. On 22 June 2022, during the green-up stage, urea (CH_4_N_2_O; 46.2% N) was manually applied in a single dose to each experimental block (Figure 1). Phenological stages of Kentucky bluegrass in the experimental field were determined according to the Descriptive Standards and Data Criteria for Poa Forage Grass Germplasm Resources [34]. The heading stage, recognized as the transition point between vegetative and reproductive growth [35], was used as the starting point for phenological sampling. Subsequent observations were conducted on 7 July (heading stage), 25 July (flowering stage), 10 August (dough stage), 21 August (maturity), and 5 September (senescence stage).

### 2.3. Measurement Indices and Methods

Within each experimental block, five 0.5 m × 0.5 m quadrats were randomly established. All individual plants within each quadrat were identified and counted as either vegetative or reproductive tillers based on the presence or absence of reproductive structures. Following the aboveground tiller survey, a soil sample measuring 0.25 m × 0.25 m in area and 0.30 m in depth was collected from each quadrat to assess the belowground bud bank (Figure 1). Samples were transported to the laboratory for processing. According to the classification principles proposed by Ott et al. [36] and Klimeš et al. [37], morphological criteria were used to distinguish viable buds from non-viable buds. Specifically, buds that were ≥2 mm long, white, and exhibited firm and turgid tissue were identified as viable buds, while buds that were brown, soft, or shriveled were considered non-viable. Buds were classified based on their position on the plant: horizontally growing rhizome apical buds, upward-growing rhizome apical buds, and rhizome node buds were collectively categorized as rhizome buds, while buds originating from basal tillering nodes were classified as tiller buds [38]. The number of each bud type was recorded accordingly.

### 2.4. Data Analysis

Prior to statistical analysis, data on belowground bud density and tiller numbers were tested for normality using the Shapiro–Wilk test and for homogeneity of variances using Levene’s test. When necessary, log or square-root transformations were applied to normalize data distributions and stabilize variances in accordance with the assumptions of parametric analyses. A two-way analysis of variance (ANOVA) was conducted within the general linear model (GLM) framework to evaluate the effects of phenological stage, nitrogen application level, and their interaction on the response variables (bud density and tiller number), with both factors treated as fixed effects. To explore the functional linkage between belowground and aboveground structures, a simple linear regression analysis was performed between belowground bud density and tiller number, and the regression model was evaluated at a 95% confidence interval. All statistical analyses were conducted using SPSS software (version 20.0, IBM Analytics, New York, NY, USA) at a significance level of *p* < 0.05, and all figures were generated using Origin software (version 2021, OriginLab Corporation, Northampton, MA, USA).

## 3. Results and Analysis

### 3.1. Interactive Effects of Nitrogen Levels and Growth Stages on Rhizome and Tiller Bud Density of Kentucky Bluegrass

**Figure 2:**
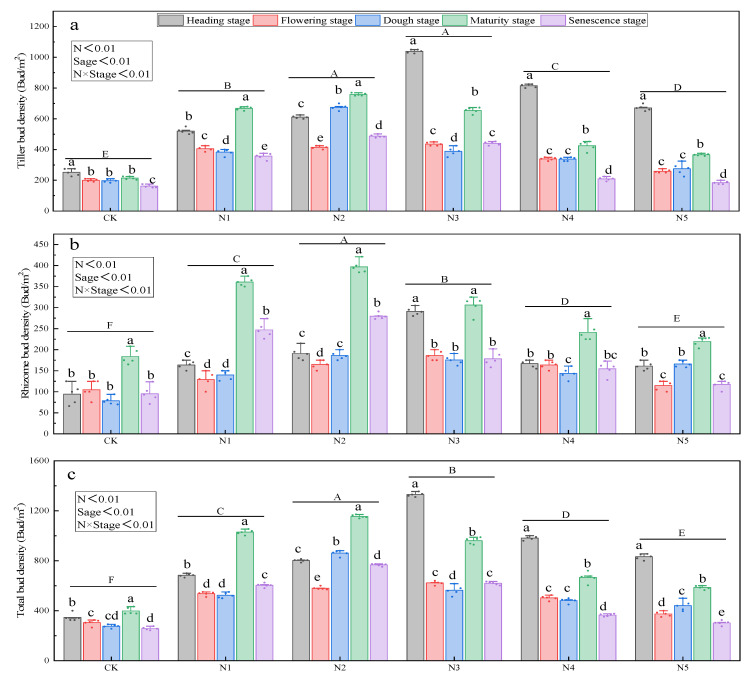
Dynamics of belowground bud density of Kentucky bluegrass under nitrogen addition conditions. Note: Lowercase letters indicate significant differences across growth stages under the same nitrogen treatment (*p* < 0.05). Bold uppercase letters above horizontal lines represent the interaction significance between nitrogen treatment and growth stage (*p* < 0.05). Nitrogen addition levels were as follows: CK = 0 g/m^2^, N1 = 6 g/m^2^, N2 = 9 g/m^2^, N3 = 12 g/m^2^, N4 = 15 g/m^2^, and N5 = 18 g/m^2^. (**a**) Effects of nitrogen addition on the seasonal dynamics of tiller bud density in Kentucky bluegrass. (**b**) Effects of nitrogen addition on the seasonal dynamics of rhizome bud density in Kentucky bluegrass. (**c**) Effects of nitrogen addition on the seasonal dynamics of total belowground bud density in Kentucky bluegrass.

Nitrogen addition, phenological stage, and their interaction had highly significant effects on the rhizome bud density, tiller bud density, and total belowground bud density of *Kentucky bluegrass* (Figure 2a–c) (*p* < 0.01). Across all the nitrogen treatments, the densities of rhizome buds, tiller buds, and total buds followed a temporal trend of initially decreasing, subsequently increasing, and then declining again, with peak values occurring at the heading and mature stages (Figure 2c). Rhizome bud density reached its maximum at the mature stage in both the nitrogen-treated and control plots. When the nitrogen input was below the N3 level, the densities of tiller buds and total buds were higher at the mature stage than at heading; however, when the nitrogen input reached or exceeded the N3 level, these densities peaked at the heading stage (Figure 2a,c) (*p* < 0.05). Throughout all the stages, rhizome bud density and total bud density were significantly higher under the N2 treatment than at other nitrogen levels (Figure 2b) (*p* < 0.05). The tiller bud density under N2 treatment did not differ significantly from that under N3 treatment, but both were higher than the levels in the remaining treatments. At the senescence stage, total bud density declined markedly across all nitrogen levels (Figure 2a–c).

The one-way ANOVA results for the effects of nitrogen addition on belowground bud density at each phenological stage are presented in Supplementary Table S1. Under nitrogen addition, every phenological stage exhibited highly significant effects (*p* < 0.01) on tiller bud density, rhizome bud density, and total belowground bud density.

### 3.2. Interactive Effects of Nitrogen Levels and Growth Stage on Aboveground Tiller Density

**Figure 3:**
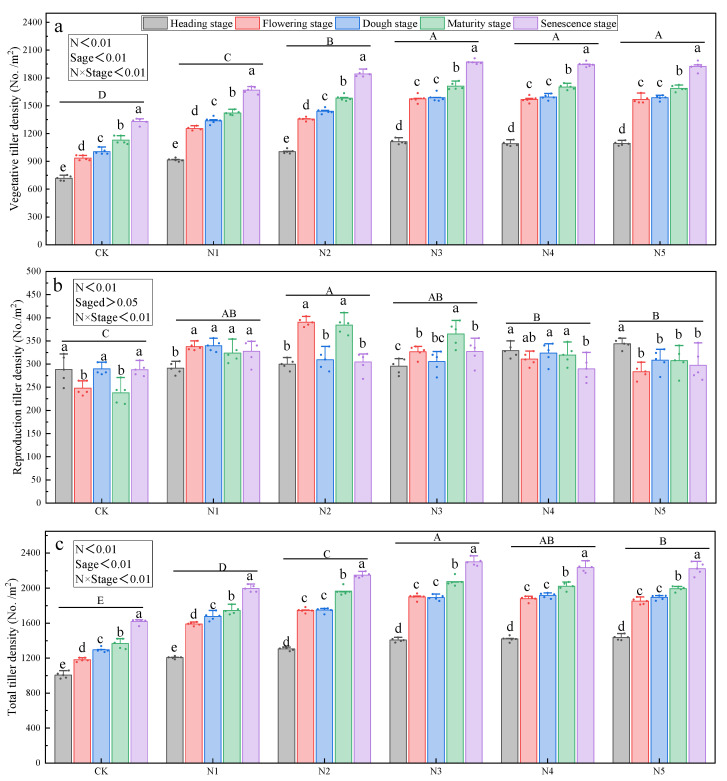
Effects of aboveground tiller number with respect to different nitrogen fertilizer gradients on Kentucky bluegrass. Note: Lowercase letters indicate significant differences across growth stages under the same nitrogen treatment (*p* < 0.05). Bold uppercase letters above horizontal lines represent the interaction significance between nitrogen treatment and growth stage (*p* < 0.05). Nitrogen addition levels were as follows: CK = 0 g/m^2^, N1 = 6 g/m^2^, N2 = 9 g/m^2^, N3 = 12 g/m^2^, N4 = 15 g/m^2^, and N5 = 18 g/m^2^. (**a**) Effects of nitrogen addition on the seasonal dynamics of vegetative tiller density in Kentucky bluegrass. (**b**) Effects of nitrogen addition on the seasonal dynamics of reproductive tiller density in Kentucky bluegrass. (**c**) Effects of nitrogen addition on the seasonal dynamics of total tiller density in Kentucky bluegrass.

Phenological stage exhibited a highly significant positive correlation with both vegetative tiller density and total tiller density (*p* < 0.01). Nitrogen addition and its interaction with phenological stage significantly affected reproductive tiller density, vegetative tiller density, and total tiller density (*p* < 0.01). Reproductive tiller density increased initially and then declined with increasing nitrogen application, reaching its maximum under the N2 treatment (Figure 3b). Under the N5 treatment, reproductive tiller density was significantly lower than that under N2 (Figure 3b) (*p* < 0.01). In contrast, vegetative tiller density and total tiller density reached a plateau under the N3 treatment and did not increase further with higher nitrogen levels (Figure 3a,c). Both were significantly higher under the N3 treatment compared to the N1 and N2 treatments (*p* < 0.01).

The one-way ANOVA results for the effects of nitrogen addition on aboveground tiller number at each phenological stage are shown in Supplementary Table S2. Under nitrogen addition, phenological stage had highly significant effects (*p* < 0.01) on vegetative tiller and total tiller densities, whereas its effect on reproductive tiller density was not significant (*p* > 0.05).

### 3.3. Correlation Between Belowground Bud Density and Aboveground Tiller Number Under Nitrogen Addition Conditions Across Phenological Stages

**Figure 4:**
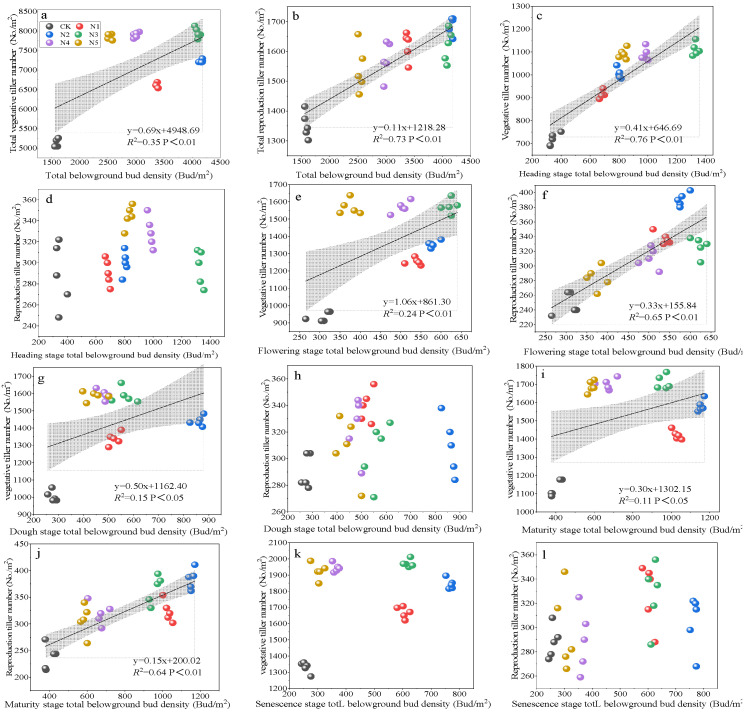
The relationships between belowground bud bank and tiller number and between tiller number and aboveground biomass in 4-year-old Kentucky bluegrass pastures under the influence of low and high nitrogen treatments. Linear regressions were fit separately for each N level. Shaded areas represent 95% confidence intervals. (**a**) Relationship between total belowground bud density and total vegetative tiller number across all phenological stages. (**b**) Relationship between total belowground bud density and total reproductive tiller number across all phenological stages. (**c**) Relationship between total belowground bud density and vegetative tiller number at the heading stage. (**d**) Relationship between total belowground bud density and reproductive tiller number at the heading stage. (**e**) Relationship between total belowground bud density and vegetative tiller number at the flowering stage. (**f**) Relationship between total belowground bud density and reproductive tiller number at the flowering stage. (**g**) Relationship between total belowground bud density and vegetative tiller number at the dough stage. (**h**) Relationship between total belowground bud density and reproductive tiller number at the dough stage. (**i**) Relationship between total belowground bud density and vegetative tiller number at the maturity stage. (**j**) Relationship between total belowground bud density and reproductive tiller number at the maturity stage. (**k**) Relationship between total belowground bud density and vegetative tiller number at the senescence stage. (**l**) Relationship between total belowground bud density and reproductive tiller number at the senescence stage.

Total belowground bud density was highly positively correlated with both reproductive tiller number and vegetative tiller number (*p* < 0.01) (Figure 4a,b). During the heading stage, vegetative tiller number increased significantly with rising bud density (R^2^ = 0.76, *p* < 0.01) (Figure 4c), while no significant relationships with reproductive tillers were observed (Figure 4d). At the flowering stage, bud density was significantly positively correlated with vegetative tiller number (R^2^ = 0.24, *p* < 0.05) (Figure 4e) and showed a highly significant positive correlation with reproductive tiller number (R^2^ = 0.65, *p* < 0.01) (Figure 4f). Belowground bud density remained strongly correlated with reproductive tiller number (R^2^ = 0.64, *p* < 0.01) and was significantly correlated with vegetative tiller number at the mature stage (R^2^ = 0.11, *p* < 0.05) (Figure 4j). However, no significant correlations were detected between bud density and either tiller type during the senescence stage (Figure 4k,l).

## 4. Discussion

### 4.1. Regulation of Belowground Bud Dynamics by Nitrogen Addition and Phenological Development in Kentucky Bluegrass

The results of this study demonstrate that nitrogen addition significantly increased the belowground bud density of Kentucky bluegrass. However, further increases in nitrogen input led to the suppression of bud density, which is consistent with our first hypothesis. This finding is in agreement with the work conducted by Dalgleish [39], who reported similar results for perennial grasses of tallgrass prairie ecosystems. Similarly, Yu [40] observed that nitrogen addition enhanced the belowground bud density of *Leymus chinensis*, while excessive nitrogen supplementation suppressed it. We found that total bud density was highest following the N2 treatment, indicating a strong positive response to moderate nitrogen input, whereas bud density decreased under the influence of high nitrogen levels (N5), suggesting that belowground bud density exhibits a nitrogen response threshold at the N2 level. Nitrogen can regulate the biosynthesis of cytokinin [19], which promotes cell division and lateral bud activation [20], thereby enhancing tillering capacity [21]. However, excessive nitrogen input (15 g/m^2^) significantly reduced total bud density, which is consistent with studies reporting suppressed clonal bud activity under nutrient oversupply conditions [40,41]. This suppression may result from a shift in carbon allocation, with more assimilates being directed toward tiller growth at the expense of root and bud development [42].

From the phenological stage perspective, belowground bud density followed a “decline–increase–decline” trend throughout the phenological stages, peaking at the heading and mature stages and declining sharply during senescence. At the heading stage, plants begin reproductive growth, during which resources are allocated not only for vegetative propagation but also seed production. This resource trade-off likely leads to a temporary reduction in bud density [43]. From the grain-filling stage to maturity, aboveground growth is largely complete, and more photosynthates are allocated to storage organs such as roots and rhizomes [36], providing sufficient energy for bud development and resulting in increased bud density. During senescence, falling temperatures and the death of aerial organs induce bud dormancy, and aging buds may undergo degradation and die [5,14,15], contributing to the overall decline in bud density.

### 4.2. Effects of Nitrogen Addition on Vegetative and Reproductive Tiller Density in Kentucky Bluegrass

Nitrogen addition significantly increased the densities of both vegetative and reproductive tillers. With an increase in nitrogen input, tiller number exhibited a trend of first rising and then declining, which supports our second hypothesis. Reproductive tiller density peaked under the N2 treatment and declined thereafter, with no significant difference between the N2 and N3 treatments. However, reproductive tiller density significantly decreased under the highest nitrogen input (N5), indicating that moderate nitrogen addition promotes sexual reproduction, whereas excessive nitrogen concentrations may suppress reproductive allocation—possibly due to excessive vegetative growth [44] or intensified resource competition [45]. This pattern is consistent with the “vegetative–reproductive trade-off” theory in regard to clonal plants [13,46]. Zhang [32] also reported that nitrogen addition reduced reproductive allocation in Poaceae species. We found that the vegetative tiller number and total tiller number peaked under the N3 treatment and plateaued with further increases in nitrogen input, indicating that additional nitrogen treatment beyond this level did not lead to a further enhancement in tiller production. This suggests N3 (12 g/m^2^) constitutes a nitrogen saturation threshold for perennial rhizomatous grasses, a finding consistent with the principle of diminishing marginal returns in grassland productivity under nitrogen enrichment conditions [47,48]. The observed plateau may be attributed to physiological saturation, wherein the nitrogen uptake and assimilation capacities of a plant are maximized and no longer responsive to additional nitrogen inputs [49].

### 4.3. Phenological Correlations Between Bud Density and Tiller Development Under Nitrogen Addition Conditions

The results of this study reveal a highly significant positive correlation between total belowground bud density and the number of both vegetative and reproductive tillers, indicating that the belowground bud bank serves as the structural foundation for tiller development in perennial grasses. The influence of bud density on vegetative tillers was primarily observed during the heading and flowering stages, and its impact on reproductive tillers was more pronounced during the flowering and maturity stages, with no significant effects detected during the senescence stage. These findings are consistent with our third hypothesis.

During the heading stage, the number of vegetative tillers increased significantly with an increase in bud density. At the mature stage, bud density remained significantly correlated with reproductive tiller number and vegetative tiller number. However, no significant correlations were observed between bud density and tiller number in the senescence stage. These findings are consistent with our third hypothesis. Vegetative tiller formation relies on early-season bud activation, constituting a critical window for tiller establishment. Similar findings were reported by Ott [36] in his study on the clonal growth dynamics of *Pascopyrum smithii* in North American grasslands. During the heading stage, plants experience peak growth and require high nutrient inputs for organ development [32]. Therefore, the demand for new vegetative tillers is highest in this stage. Belowground bud density showed a significant positive correlation with vegetative tiller number and reproductive tiller number at the flowering stage. This finding may be attributed to the continued functional role of vegetative tillers in supporting seed development through photosynthetic activity. Some buds possess reproductive potential in clonal perennials but require higher activation thresholds and hormonal signals—such as gibberellins and cytokinins—to initiate reproductive differentiation [15,50,51]. These signals intensify around flowering [52,53], facilitating reproductive bud expression. Although most nutrients are allocated for seed filling during these stages, buds that were previously activated may manifest as reproductive tillers with a temporal lag [1]. This late-stage emergence of reproductive tillers may represent a “reproductive insurance strategy” that compensates for seed loss or enhances reproductive output under favorable conditions [36]. According to the reproductive allocation theory, plants shift resources from vegetative growth to reproductive output in the latter part of their life cycles [54,55]. This trade-off is particularly evident in alpine, arid, or short-growing-season environments, where resources are preferentially allocated to flowering and seed production [56,57,58]. Tiller differentiation ceased and aboveground structures stabilized during the senescence stage. Resource input declined, photosynthetic organs began to senesce, and assimilates were increasingly allocated to storage organs [59]. Although the belowground bud bank remained present, its activity was suppressed, and high bud density no longer translated into increased tiller formation.

## 5. Conclusions

This study demonstrates that 9 g/m^2^ (N2) is the optimal nitrogen threshold for promoting belowground bud bank formation in Kentucky bluegrass, with the highest total, rhizome, and tiller bud densities observed at this level. Higher nitrogen inputs inhibited bud development and reduced potential productivity. Nitrogen addition showed a diminishing marginal effect on vegetative tiller and total tiller numbers, with no significant increase beyond 12 g/m^2^ (N3), while reproductive tillers declined beyond N2, indicating the negative effects of excessive nitrogen. The regulation of shoot development by bud density was phenology-dependent: vegetative tillers were most responsive at the heading stage, while reproductive tillers were most responsive upon reaching maturity, and the correlations weakened during senescence. These results highlight the joint role of nitrogen input and phenological stage in shaping bud–shoot dynamics.

## Data Availability

The datasets generated for this study are available upon request from the corresponding author.

## Supplementary Materials

The following supporting information can be downloaded at: https://www.mdpi.com/article/10.3390/plants14142164/s1, Table S1. One-way ANOVA for the effect of nitrogen application on belowground bud density at each phenological stage. Table S2. One-way ANOVA for the effect of nitrogen application on aboveground tiller number at each phenological stage.
