# Supplementary material for: Phenological Stage and Nitrogen Input Coordinately Regulate Bud Bank Dynamics and Shoot Allocation in an Alpine Clonal Perennial Grass
Source: Plants (Basel). 2025 Jul 14;14(14):2164. doi: 10.3390/plants14142164 (PMC12298651; doi:10.3390/plants14142164)
Supplement: Supplementary file 1 [file plants-14-02164-s001.zip › plants-3704400-supplementary.docx]

**Table S1.** One-way ANOVA for the effect of nitrogen application on belowground bud density at each phenological stage

| phenological stage | Tiller bud density | | Rhizome bud density | | Total bud density | |
| --- | --- | --- | --- | --- | --- | --- |
|  | F | *P* | F | *P* | F | *P* |
| Heading stage | 1346.467 | ＜0.01 | 104.956 | ＜0.01 | 1392.994 | ＜0.01 |
| Flowering stage | 366.396 | ＜0.01 | 24.626 | ＜0.01 | 241.562 | ＜0.01 |
| Dough stage | 246.974 | ＜0.01 | 67.184 | ＜0.01 | 242.37 | ＜0.01 |
| Maturity stage | 828.81 | ＜0.01 | 130.627 | ＜0.01 | 661.641 | ＜0.01 |
| Senescence stage | 613.074 | ＜0.01 | 108.311 | ＜0.01 | 1167.6 | ＜0.01 |

**Table S2.** One-way ANOVA for the effect of nitrogen application on aboveground tiller number at each phenological stage

| phenological stage | Vegetative tiller density | | Reproductive tiller density | | Total tiller density | |
| --- | --- | --- | --- | --- | --- | --- |
|  | F | *P* | F | *P* | F | *P* |
| Heading stage | 192.165 | ＜0.01 | 8.669 | 0.138 | 198.424 | ＜0.01 |
| Flowering stage | 312.802 | ＜0.01 | 73.108 | 0.128 | 455.322 | ＜0.01 |
| Dough stage | 242.833 | ＜0.01 | 3.694 | 0.062 | 272.085 | ＜0.01 |
| Maturity stage | 223.722 | ＜0.01 | 24.181 | 0.757 | 169.988 | ＜0.01 |
| Senescence stage | 250.276 | ＜0.01 | 2.455 | 0.527 | 149.433 | ＜0.01 |
